# A novel colorectal cancer test combining microsatellite instability and *BRAF/RAS* analysis: Clinical validation and impact on Lynch syndrome screening

**DOI:** 10.1038/s44276-024-00072-8

**Published:** 2024-07-01

**Authors:** Richard Gallon, Patricia Herrero-Belmonte, Rachel Phelps, Christine Hayes, Elizabeth Sollars, Daniel Egan, Helena Spiewak, Sam Nalty, Sarah Mills, Peh Sun Loo, Gillian M. Borthwick, Mauro Santibanez-Koref, John Burn, Ciaron McAnulty, Michael S. Jackson

**Affiliations:** 1 Translational and Clinical Research Institute, Faculty of Medical Sciences, Newcastle University, Newcastle upon Tyne, UK; 2 Northern Genetics Service, The Newcastle upon Tyne Hospitals NHS Foundation Trust, Newcastle upon Tyne, UK; 3 Biosciences Institute, Faculty of Medical Sciences, Newcastle University, Newcastle upon Tyne, UK; 4 North West Genomic Laboratory Hub, Manchester Centre for Genomic Medicine, Manchester University NHS Foundation Trust, Manchester, UK; 5 North East and Yorkshire Genomic Laboratory Hub Central Lab, St James’s University Hospital, Leeds, UK; 6 Sheffield Diagnostic Genetics Service, North East and Yorkshire Genomic Laboratory Hub, Sheffield Children’s NHS Foundation Trust, Sheffield, UK; 7 Northumbria Healthcare NHS Foundation Trust, Newcastle upon Tyne, UK; 8 Department of Cellular Pathology, Royal Victoria Infirmary, The Newcastle Upon Tyne Hospitals NHS Foundation Trust, Newcastle upon Tyne, UK

## Abstract

**Background:**

Lynch syndrome (LS) is under-diagnosed. UK National Institute for Health and Care Excellence guidelines recommend multistep molecular testing of all colorectal cancers (CRCs) to screen for LS. However, the complexity of the pathway has resulted in limited improvement in diagnosis.

**Methods:**

One-step multiplex PCR was used to generate sequencing-ready amplicons from 14 microsatellite instability (MSI) markers and 22 *BRAF*, *KRAS*, and *NRAS* mutation hotspots. MSI and *BRAF/RAS* variants were detected using amplicon-sequencing and automated analysis. The assay was clinically validated and deployed into service in northern England, followed by regional and local audits to assess its impact.

**Results:**

MSI analysis achieved 99.1% sensitivity and 99.2% specificity and was reproducible (r = 0.995). Mutation hotspot analysis had 100% sensitivity, 99.9% specificity, and was reproducible (r = 0.998). Assay-use in service in 2022–2023 increased CRC testing (97.2% (2466/2536) versus 28.6% (601/2104)), halved turnaround times, and identified more CRC patients at-risk of LS (5.5% (139/2536) versus 2.9% (61/2104)) compared to 2019–2020 when a multi-test pathway was used.

**Conclusion:**

A novel amplicon-sequencing assay of CRCs, including all biomarkers for LS screening and anti-EGFR therapy, achieved >95% testing rate. Adoption of this low cost, scalable, and fully automatable test will complement on-going, national initiatives to improve LS screening.

## Introduction

Lynch syndrome (LS) is an autosomal dominant cancer predisposition syndrome caused by germline pathogenic variants (PVs) affecting one of four mismatch repair (MMR) genes: *MLH1*, *MSH2*, *MSH6*, and *PMS2*. These PVs include germline 3’ deletions in the *EPCAM* gene that lead to hypermethylation of surrounding DNA, including the downstream promoter of *MSH2* causing *MSH2* silencing. LS predisposes individuals to the development of a spectrum of malignancies including gastrointestinal tract cancers, predominantly colorectal cancer (CRC), endometrial cancer (EC), and genitourinary tract cancers, with a median age of onset of 45-60 years. The tumour spectrum and disease penetrance depend on which MMR gene is affected [1]. LS carriers can benefit from risk reducing surgery, such as colectomy and hysterectomy, cancer surveillance, such as colonoscopy, and daily aspirin intake to approximately halve their CRC risk [2, 3].

The MMR system ensures fidelity of DNA replication by detecting and initiating repair of base mismatches and short insertion-deletion loops [4, 5]. Loss of MMR and the resulting genomic instability can promote tumorigenesis [6, 7] and MMR deficiency is found in many different tumour types both sporadically and in association with LS [8]. MMR deficient cancers are also associated with resistance to some chemotherapeutic agents, response to immune checkpoint inhibitors (ICIs), and, in the context of early stage CRC, better prognosis [9].

In 2017, the UK National Institute of Health and Care Excellence (NICE) published Diagnostics Guidance 27 recommending all CRC patients should be screened for LS. Tumours should be reflexively tested for MMR deficiency, with MMR deficient tumours further tested for *MLH1* promoter methylation and/or *BRAF* c.1799T>A (p.V600E) to exclude sporadic MMR deficient CRCs from the screening pathway. At-risk patients should then be offered genetic counselling and germline genetic testing [10]. The major barriers to tumour testing for LS screening include financial and staff resourcing [11–13], and an audit of LS screening across England in 2019 by the NHS National Disease Registration Service (NDRS) found that only 44% of CRCs were tested for MMR deficiency and that complete tumour testing took a median 58 days [14].

Tumour MMR deficiency tests include immunohistochemistry (IHC) to detect loss of MMR protein expression, and fragment length analysis (FLA) of PCR amplicons to detect microsatellite instability (MSI), the accumulation of insertion-deletion variants in tandem repeat DNA sequences (microsatellites). IHC and FLA are widely available, are highly sensitive and specific, and have a modest cost, but their scalability to high throughput LS screening is limited by the availability of expert personnel to interpret the results and the need for subsequent tumour tests to increase screening specificity [15, 16]. Innovative methodologies to streamline LS screening are needed. Nearly a decade ago, it was shown that MSI status can be determined from microsatellites covered by tumour gene panel, exome, and/or genome sequencing, in parallel with other genetic biomarkers such as *BRAF* c.1799T>A [17–19]. However, sequencing-based LS screening has not been adopted in the UK due, in part, to cost and data burden [20].

Previously, we developed a low cost and fully automatable, molecular inversion probe (MIP) and amplicon sequencing assay that multiplexes MSI and *BRAF* analyses in a single CRC molecular test for LS screening [10] and ICI therapy [21]. It also incorporated *RAS* mutation hotspots to inform anti-EGFR therapy [22], and was clinically validated for service in the Newcastle upon Tyne Hospitals NHS Foundation Trust in October 2020 ref. [23]. However, ~14% of DNA samples were of too low quantity or quality to be assayed, necessitating a salvage, multi-test pathway. Here, we describe the clinical validation of a simpler, one-step multiplex PCR implementation of the assay, which both resolves this issue and incorporates more sensitive MSI markers, to provide a single test applicable to CRC biopsies for LS screening and oncological management. We also analyse the impact of these amplicon sequencing assays on clinical service in the North East of England and Cumbria region.

## Materials and methods

### Samples

The Newcastle MSI-Plus Assay was developed and validated using the following samples and reference methods (Supplementary Table S1).

Excess diagnostic DNA samples from formalin-fixed paraffin embedded (FFPE) CRCs (*n* = 238) and peripheral blood leucocytes (PBLs) (*n* = 1) were sourced from the Northern Genetics Service (The Newcastle upon Tyne Hospitals NHS Foundation Trust) for assay development and clinical validation. Testing referral required FFPE CRCs to have a minimum 20% tumour cell content. Ethical approval for use of excess diagnostic samples during assay development was granted by the NHS Health Research Authority (REC reference 13/LO/1514). Patient consent was obtained for use of excess diagnostic samples for clinical validation of novel assays. External Quality Assessment (EQA) FFPE CRC DNA samples (UK NEQAS) provided additional controls.

Samples to simulate different tumour content were created by mixing HCT116 CRC cell line (COSMIC ID: COSS905936) and PBL DNA. Empirical estimates of HCT116 content were calculated using the sequencing read frequencies of two SNP alleles (rs2283006, rs7905384) found in HCT116 but not the PBL DNA. The Tru-Q 4 (5% Tier) Reference Standard (Horizon Discovery, Cambridge, UK) was used to assess assay detection limits for mutation hotspot variants. Sample DNA concentrations (ng/μl) were measured by QuBit 3 Fluorometer (Invitrogen, Waltham, MA, USA). Sample dilutions used de-ionised water. Sample purification used AMPure XP beads (Beckman Coulter, Brea, CA, USA).

MIP amplicon sequencing or the MSI Analysis System v1.2 (Promega, Madison, WI, USA), without paired normal tissue, provided MSI reference results. CRCs classified as MSI-high (MSI-H) due to instability in only 2/5 MSI markers of the MSI Analysis System v1.2 were often discordant with MMR IHC and the MSI-Plus assay (Supplementary Fig. S1) and were only included if an alternative reference was available to be used instead, including either IHC of MMR protein expression or MIP amplicon sequencing. The MassARRAY System (Agena Bioscience, San Diego, CA, USA) and/or MIP amplicon sequencing [23] provided *KRAS* and *NRAS* reference results. High resolution melt curve analysis [24], the MassARRAY System (Agena Bioscience, San Diego, CA, USA), and/or MIP amplicon sequencing [23] provided *BRAF* reference results.

### One-step multiplex PCR primers

Primer designs were adapted from those described by Ciosi et al. [25]. Target-specific primers were generated using PrimerBLAST [26] and had sequencing adapters from MIP amplicon sequencing [27] added to the 5’ end. Primer designs were checked for interactions using Multiplex Manager with default settings [28], and used if multiplex PCR amplification and sequencing achieved read counts ≥10% of the median read depth across targets. The final multiplex includes 14 primer pairs targeting 14 highly sensitive MSI markers selected from CRC exome sequence data [29] or PBL genome sequence data from individuals with constitutional mismatch repair deficiency (CMMRD) [30], and 7 primer pairs targeting 22 *BRAF*, *KRAS*, and *NRAS* mutation hotspot positions (Supplementary Table S2). Separate multiplex primer pools were prepared for each sample index (nGS Oligos, Metabion, Planegg, Germany): The 42 primers (21 pairs) were mixed in equal volume (unless indicated otherwise) and then diluted to a final 1 µM concentration for each primer using 10 mM Tris Hydrochloride pH8.0 (Fisher BioReagents, Waltham, MA, USA).

### Multiplex PCR

Multiplex PCR amplification used 1x HS VeriFi Buffer (PCR Biosystems, London, UK), 0.5U HS VeriFi Polymerase (PCR Biosystems, London, UK), 1 μl of multiplex primer pool (1 pmol of each primer), and a target range of 1–100 ng of sample DNA in a total reaction volume of 25 μl. Reactions were incubated in a thermocycler for 60 seconds at 95 °C, followed by 30 cycles of 30 seconds at 95 °C, 90 seconds at 57 °C, and 60 seconds at 72 °C, followed by 120 seconds at 72 °C. Amplicons were visualised using gel electrophoresis (Supplementary Fig. S2).

### Amplicon sequencing, variant calling, and MSI classification

Amplicon libraries were prepared and sequenced to a target read depth of 2000–3000x as previously described [23]. FASTQ reads were aligned to human reference genome hg19 using BWA mem [31]. SAM files were converted to BAM format using SAMtools view, sort, and index [32]. Reads containing a PCR artefact due to *KRAS* and *NRAS* sequence homology, causing *KRAS* c.37G>A false positive calls, were identified by a definitive sequence and removed from alignment files using Picard (https://broadinstitute.github.io/picard/). Variant and wild type sequence counts were summarised from (artefact-free) SAM files using R (version 4.2.2, https://www.r-project.org/) [29]. Read down-sampling to simulate low read depths used R.

Mutation hotspot variant allele frequencies (VAFs) were calculated by dividing the number of variant allele reads by the total number of reads covering the position. MSI status was determined by calculating an MSI score following a naïve Bayesian method. In brief, a training cohort of samples of known MSI status is used to determine the distribution of deletion allele frequencies and deletion allelic bias in each MSI marker for MSI-H CRCs and microsatellite stable (MSS) CRCs. Observations from a test sample can then be compared to these training cohort distributions to determine the probability the test sample is MSI-H and the probability the test sample is MSS. The a priori probabilities a test sample is MSI-H and MSS are set at 0.15 and 0.85, respectively. An MSI score for a test sample is the decadic logarithm of the probability it is MSI-H divided by the probability it is MSS. For details, please see Redford et al. [29]. MSI markers with <50 reads were excluded from MSI score calculation. An MSI score >0 indicates the sample is MSI-H (equivalent to MMR deficient), an MSI score <0 indicates the sample is MSS (equivalent to MMR proficient), and an MSI score of 0 is inconclusive.

### Clinical audit

Clinical audits were conducted during November-December 2023 following The Caldicott Principles (https://www.gov.uk/government/publications/the-caldicott-principles). Audit data covered one year periods from 18th October to 17th October for 2019–2020, 2021–2022, and 2022–2023, corresponding to the use of three different CRC testing strategies.

The regional audit to determine the number of CRC registrations was conducted by the NHS Northern Cancer Alliance using Rapid Cancer Registration Data from the NDRS. The counts of CRC registrations are approximations based on proxy data such as registered events in the cancer patient pathway (e.g. surgery appointments) and used one year periods running from October 1st to September 30th rather than the dates specified for all other audits.

The regional audit of CRC molecular and germline genetic testing used the Northern Genetics Service test database.

The audit of CRC registrations and of CRC molecular and germline genetic testing in the Northumbria Healthcare Trust used data collected prospectively (by author SM) with complete ascertainment being ensured through triangulation of sources, including but not limited to the patient administration and pathology reporting systems and weekly multi-disciplinary meeting lists.

### Statistical analyses and graphics

Statistical analyses and graphics used R (version 4.2.2, https://www.r-project.org/) and package ggplot2 [33]. Confidence intervals (CI) for sensitivity and specificity were calculated from a binomial distribution. Pearson’s r or Spearman’s ρ correlation tests were used assuming a linear or monotonic relationship, respectively. The Mann-Whitney U test was used to compare continuous variables between two groups. Fisher’s exact test was used to compare frequencies between groups. Localised regression used the default LOESS (locally estimated scatterplot smoothing) model of geom_smooth from the ggplot2 R package. Statistical tests were two-sided.

## Results

### Clinical validation: MSI analysis

#### Sensitivity and specificity

**Fig. 1:**
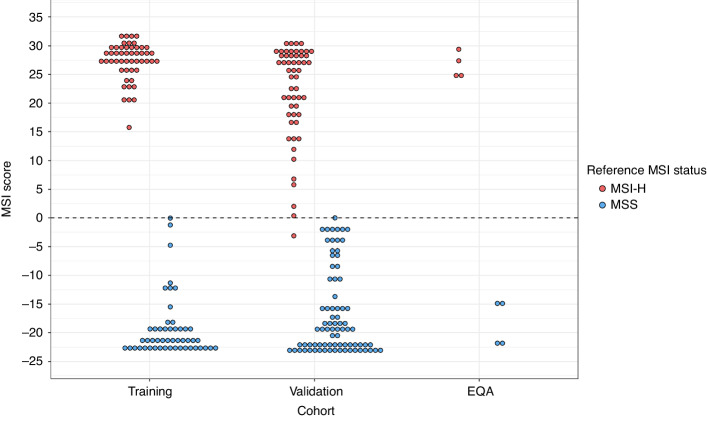
MSI scores of CRCs used to train and validate the MSI classifier of the Newcastle MSI-Plus Assay. An MSI score >0 indicates the sample is MSI-H, and an MSI score <0 indicates the sample is MSS. The reference MSI status was determined by the MSI Analysis System v1.2 (Promega) or, where the MSI Analysis System v1.2 result was uncertain, immunohistochemistry and/or MIP amplicon sequencing.

The MSI classifier of the Newcastle MSI-Plus Assay uses a naïve Bayesian method to calculate the relative probability a sample is MSI-H or MSS and requires training on sequence data from samples of known MSI status [29]. An MSI score >0 indicates a higher probability the sample is MSI-H, whilst an MSI score <0 indicates a higher probability the sample is MSS. Fifty MSI-H and 50 MSS FFPE CRCs were assayed to train the MSI classifier. This training cohort was classified with 100.0% sensitivity (50/50; 95% CI: 92.9–100.0%) and 100.0% specificity (50/50; 95% CI: 92.9–100.0%) (Fig. 1). An independent validation cohort of 55 MSI-H and 83 MSS FFPE CRCs and 4 MSI-H and 4 MSS EQA CRCs was then assayed. Six samples (1 MSI-H, 5 MSS) had read depths too low to generate an MSI score. Excluding these, the validation cohort was classified with 98.3% sensitivity (57/58; 95% CI: 90.8–100.0%) and 98.8% specificity (81/82; 95% CI: 93.4–100.0%). One discordant sample was classified as MSS (MSI score -3.1) in contrast to its reference MSI-H status. The other had an inconclusive classification (MSI score 0.0) and reference MSS status (Fig. 1).

#### Quality control metrics

**Fig. 2:**
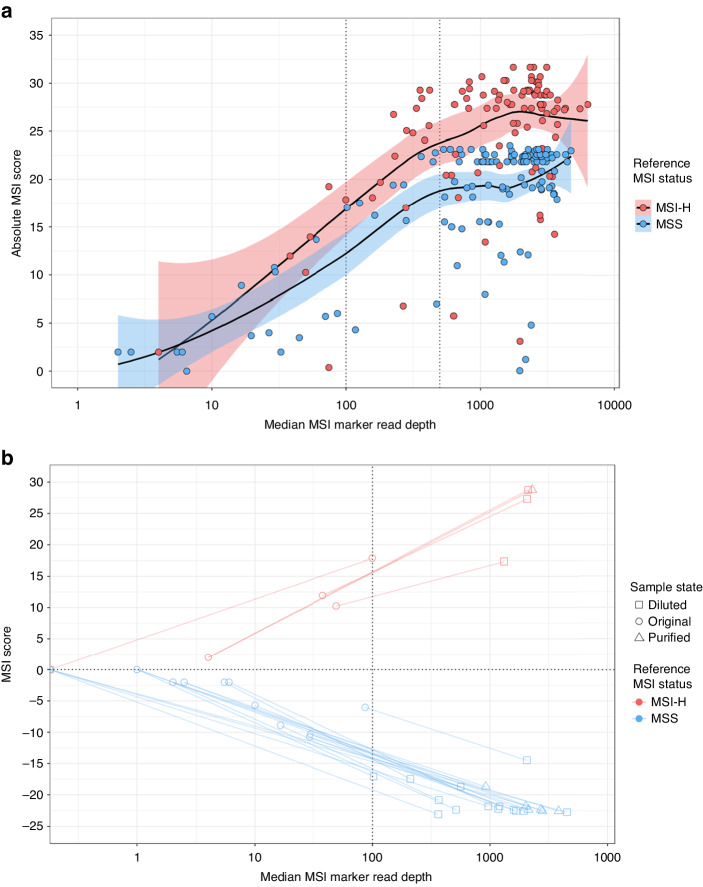
Median MSI marker read depth as an assay quality control metric, and the impact of repeat testing poor quality samples. **a** Absolute MSI score compared to median MSI marker read depth of training and validation cohort samples analysed by the Newcastle MSI-Plus Assay. The localised regression lines include shading, coloured by reference MSI status, to represent 95% confidence intervals. The vertical dotted lines represent a median MSI marker read depth of 100 or of 500. **b** The effect of sample dilution and/or purification on MSI score and median MSI marker read depth of samples that initially failed quality control (median reads per MSI marker <100). The lines connect original sample data to diluted and/or purified sample data.

The relationship between MSI score and MSI marker read depth was assessed to define read depth quality control (QC) thresholds. Across both CRC cohorts, sample median MSI marker read depths correlated with absolute MSI scores (ρ = 0.401; *p* = 1.12 × 10^–10^) and the localised regression showed a plateau in absolute MSI score at read depths ≥500 (Fig. 2a). A QC threshold of median MSI marker read depth ≥500 was deemed too stringent, with 55/246 (22.4%) CRCs falling below this. At a less stringent threshold of median MSI marker read depth ≥100, only 30/246 (12.2%) CRCs failed QC. There was no significant association between sample DNA input quantity and QC failure (*p* = 0.097), but 16/30 (53.3%) had exceptionally high mutation hotspot read depths that were ≥5-fold higher than those of MSI markers (Supplementary Fig. S3), suggesting selective inhibition of MSI marker amplification. Fifteen of these 16 samples, plus three additional samples with a median MSI marker read depth of 0, had residual DNA available for further testing. All 18 were diluted 5-fold and seven had sufficient material to also be purified (see methods). Diluted and purified samples were assayed and all but one gave increased MSI marker read depths, passed the QC threshold of median MSI marker read depth ≥100, and had more extreme MSI scores in accordance with known MSI status (Fig. 2b), consistent with a sample quality issue. This suggested that the read depth ratio between MSI markers and mutation hotspots is a useful indicator of sample quality and that repeat testing of samples initially failing QC would have a high success rate.

#### Reproducibility and tumour content detection limit

**Fig. 3:**
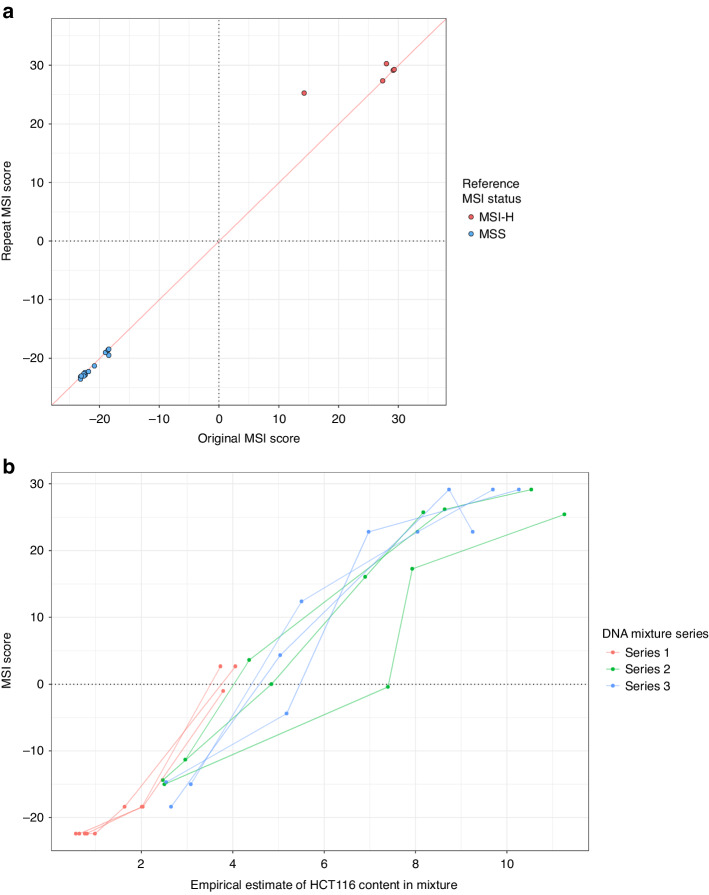
MSI score reproducibility and tumour content detection limit of the Newcastle MSI-Plus Assay. **a** Comparison of MSI scores between repeat and original assays of 24 CRCs from the validation cohort—one repeated sample is not shown as it did not have sufficient reads to generate an MSI score. **b** MSI scores of mixtures of HCT116 cell line DNA with peripheral blood leukocyte DNA in different mix ratios. The empirical estimate of HCT116 content in the mixture was calculated based on allele frequencies of two SNP alleles found in HCT116 not present in the blood DNA.

To assess reproducibility, 6 MSI-H and 19 MSS CRCs were repeat assayed. The median MSI marker read depths were correlated between repeats, with one sample failing QC in both tests (r = 0.738, *p* = 2.6 × 10^-5^; Supplementary Fig. S4). The MSI scores were strongly correlated between repeats (r = 0.995; *p* < 1.0 × 10^–15^; Fig. 3a), demonstrating MSI analysis is reproducible. Testing mixtures of MSI-H cell line HCT116 DNA and non-neoplastic PBL DNA, to simulate different tumour cell content, showed the assay can reliably classify MSI status from ≥10% tumour cell content (Fig. 3b), below the minimum 20% requested for clinical testing.

### Clinical validation: mutation hotspot analysis

#### Variant allele frequency threshold and quality control

To call *BRAF*, *KRAS*, and *NRAS* variants, VAF and read depth thresholds needed to be established that are robust to technical variation. A VAF of 0.05 (5%) is generally accepted as clinically relevant and it has been suggested that ≥250 reads are sufficient for accurate somatic variant calling of VAFs ≥0.05 ref. [34]. VAF detection at different read depths was explored using the Tru-Q 4 (5% Tier) Reference Standard (Horizon), which contains seven *BRAF*, *KRAS*, and *NRAS* variants, four of which have VAFs of 0.05.

**Fig. 4:**
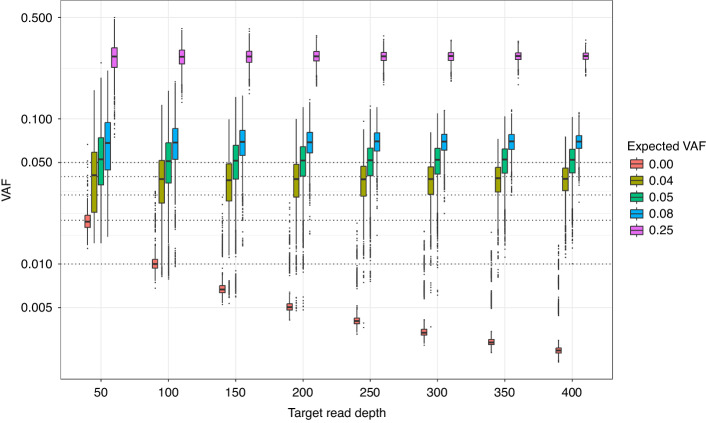
Simulating the effect of mutation hotspot read depth on variant allele frequency. The mutation hotspot variant allele frequencies (VAFs) at different simulated read depths were calculated following down sampling of reads from the triplicate analysis of the Tru-Q sample. Variants are grouped by the expected VAF. Reads for each mutation hotspot position were sampled 1000 times for each simulated target read depth. The y-axis uses a decadic logarithmic scale and, hence, VAFs of 0.00 (*n* = 1,393,440 data points) are excluded from the plot. The dotted lines represent VAFs of 0.01, 0.02, 0.03, 0.04, and 0.05.

The Tru-Q sample was first assayed in triplicate to the standard 2000–3000x target read depth. All variants were detected with VAFs similar to expected (r = 0.992; *p* < 1 × 10^–15^; Supplementary Fig. S5). The Tru-Q sequencing reads were then down-sampled to simulate reads depths from 50-400x, with 1000 simulations per target read depth for each replicate. Decreasing read depth was associated with increased allele frequency error. At a sampling depth of ≥250 reads and using a VAF ≥ 0.01 threshold for variant calling, there were 71 (0.04%) false positive variant calls and 14 (0.12%) false negative variant calls for variants with expected 0.05 VAF (Fig. 4). Hence, these thresholds were used for variant calling when assessing assay performance.

#### Sensitivity, specificity, and reproducibility

**Fig. 5:**
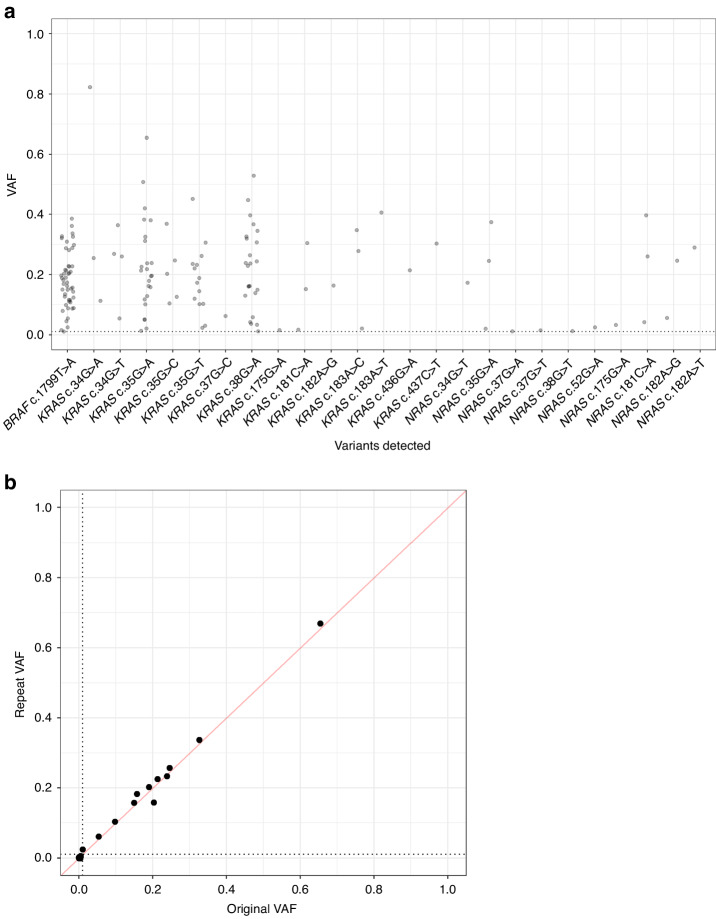
The allele frequencies and reproducibility of mutation hotspot variant calling by the Newcastle MSI-Plus Assay. **a** Variant allele frequencies (VAF) of *BRAF*, *KRAS*, and *NRAS* variants detected by the Newcastle MSI-Plus Assay in the training and validation cohort CRCs (*n* = 238) with a minimum VAF of 0.01 (dotted line) and supported by ≥250 reads. **b** Comparison of VAFs detected between repeat and original assays of 25 CRCs from the validation cohort. The dotted lines represent VAF = 0.01.

The 238 FFPE CRCs of the training and validation cohorts had 49 *BRAF*, 86 *KRAS*, and 15 *NRAS* variants detected with VAF ≥ 0.01 and supported by ≥250 reads (Fig. 5a). Of note, more CRCs had <250 reads for the *BRAF* amplicon than for *RAS* amplicons (63 vs 7–32; *p* = 5.0 × 10^–4^; Supplementary Table S3). Therefore, for clinical service, the primer multiplex was reconfigured with 1.5x relative primer concentration for *BRAF* to increase read depths. Despite this, there were sufficient samples with ≥250 reads and a reference method available to estimate assay accuracy across each mutation hotspot position. Overall, considering the three possible substitution variants at each position, the assay achieved 100% sensitivity (80/80 variants called of variant-positive reference results; 95% CI: 95.5–100.0%) and 99.9% specificity (6259/6265 variants not called of variant-negative reference results; 95% CI: 99.8–100.0%). The six false positive variants had low VAFs of 0.010–0.051 and could be PCR and/or sequencing error or could be below the detection limits of the reference methods (Supplementary Table S3). The CRCs that were repeat assayed to determine reproducibility of MSI analysis (*n* = 25) had 5 *BRAF*, 6 *KRAS*, and 1 *NRAS* variants called with a strong correlation in VAF between original and repeat assays (r = 0.998, *p* < 10^–15^) (Fig. 5b), demonstrating variant calling is reproducible.

### Service impact

On 18th October 2022, the Newcastle MSI-Plus Assay was deployed into clinical service in the Northern Genetics Service laboratory of The Newcastle upon Tyne Hospitals NHS Foundation Trust (and part of the North East and Yorkshire Genomic Laboratory Hub), providing testing for seven pathology laboratories covering the North East of England and Cumbria region. To pass QC, samples require a median MSI marker read depth ≥100 and ≥250 reads per mutation hotspot position. Samples failing any QC metric are repeat tested, with any samples with a median read depth ratio ≥5 for mutation hotspot versus MSI marker amplicons first being diluted or purified. MSI scores between 5 and -5 require repeat testing, with the classification being accepted if repeat scores are concordant, and the sample failing QC if scores are discordant. Based on these criteria, ~95% of samples pass QC on the first test (Supplementary Fig. S6). Clinical validation suggested the assay can call mutation hotspot variants accurately using a ≥ 0.01 VAF threshold, but only VAFs ≥ 0.04 are reported due to the uncertain clinical relevance of low frequency variants and to conservatively avoid false positives that would make a patient ineligible for germline LS testing or anti-EGFR therapy. Reports include assay results and recommendations on LS risk [10], ICI therapy [21], and anti-EGFR therapy [22].

In one year of regional service, 2565 samples from 2466 CRC patients were referred for testing. 344 patients (13.9%) had an MSI-H CRC, and 139 patients (5.64%) were at-risk of LS due to an MSI-H and *BRAF* wild type CRC (Table 1). Overall, 1015 patients (41.2%) had a CRC with a *KRAS* variant, 81 (3.3%) had an *NRAS* variant (6 [0.2%] also had a *KRAS* variant), and 344 (13.9%) had a *BRAF* variant (2 [0.1%] also had a *RAS* gene variant).

**Table 1:** Audit of LS screening in CRC patients according to NICE Diagnostics Guidance 27 ref. [10] across three years of service representing different testing methods at the Northern Genetics Service, The Newcastle upon Tyne Hospitals NHS Foundation Trust.

	Month of CRC test activation	CRCs tested, sample n	Mean CRC test TAT, working days	Median CRC test TAT, working days	CRCs tested, patient n (% CRC diagnoses)**	MSI-H CRCs, patient n (% of tested)	MSI-H + *BRAF* WT CRCs, patient n (% of tested)	Germline tested patient n (% of eligible patients)***	Mean germline test TAT from CRC test activation, working days	Median germline test TAT from CRC test activation, working days	Germline LS diagnosis, patient n (% of tested)
**Newcastle MSI-Plus Assay 18th Oct. 2022 - 17th Oct. 2023**	October >18th	103	7.5	7	101	14	5	2	162	162	0
	November	258	7.5	7	249	43	15	9***	114	104	2
	December	224	6.1	6	215	26	10	7	142	144	2
	January	231	6.2	6	222	27	15	6	109	102	1
	February	165	6.2	6	156	24	13	5	133	151	0
	March	231	5.8	6	222	28	11	3	125	122	1
	April	184	5.6	5	179	14	5	1	136	136	0
	May	255	5.4	5	246	36	12	2	88	88	0
	June	200	5.8	5	193	31	12	1	83	83	0
	July	221	8.6	8	207	39	16	1	69	69	0
	August	199	6.4	6	191	34	12	0	NA	NA	0
	September	194	6.3	6	187	19	9	0***	NA	NA	0
	October <17th	100	6.4	6	98	9	4	0	NA	NA	0
	**Total**	**2565**	**6.4**	**6**	**2466 (97.2%)**	**344 (13.9%)**	**139 (5.6%)**	**37 (26.6%)**	**121**	**135**	**6 (16.2%)**
**MIP Amplicon Sequencing or MSI by FLA and** ***BRAF*** by MassARRAY* 18th Oct. 2021 - 17th Oct. 2022	October >18th	219	8.2	11	55	8	4	2	147	147	1
	November	199	8.2	11	119	15	8	3	261	182	1
	December	197	8.5	8	166	20	7	5	179	156	1
	January	179	9.1	8	212	22	9	4	195	220	0
	February	220	8.1	8	191	19	8	3	204	183	0
	March	179	9.8	7	188	22	10	3	174	169	1
	April	213	9.3	8	166	22	12	4	164	177	2
	May	208	8.4	7	207	17	7	4	220	225	2
	June	252	10.5	9	174	24	14	4	164	159	0
	July	116	7.7	9	203	23	8	3	187	181	2
	August	57	11.6	8	199	24	13	6	175	161	2
	September	133	11.1	10	244	34	13	10	173	156	4
	October <17th	178	8.5	7	112	17	9	3	120	83	1
	**Total**	**2350**	**9.0**	**8**	**2236 (95.1%)**	**267 (11.9%)**	**122 (5.5%)**	**54 (44.3%)**	**181**	**172**	**17 (31.5%)**
**MSI by FLA and** ***BRAF*** by MassARRAY* 18th Oct. 2019 - 17th Oct. 2020	October >18th	23	14.3	12	21	6	3	3	239	228	1
	November	38	13.5	10	38	8	4	3	188	187	0
	December	27	11.5	10	27	5	0	0	NA	NA	0
	January	26	14.8	13	26	6	2	0	NA	NA	0
	February	56	13.9	11	54	14	6	2	307	307	2
	March	60	19.2	16	57	18	8	3	245	150	1
	April	32	19.2	19	31	13	8	2	120	120	0
	May	44	14.8	14	42	10	6	1	135	135	0
	June	44	10.5	10	42	8	3	1	116	116	0
	July	65	15.4	10	64	17	7	4	199	203	1
	August	61	15.2	15	58	13	2	1	402	402	0
	September	92	10.5	10	89	20	10	4	184	184	2
	October <17th	54	8.7	8	52	10	2	1	751	751	0
	**Total**	**622**	**13.8**	**12**	**601 (28.6%)**	**129 (21.5%)**	**61 (10.2%)**	**25 (41.0%)**	**232**	**187**	**7 (28.0%)**

*CRC* colorectal cancer, *FLA* fragment length analysis, *LS* Lynch syndrome, *TAT* turnaround time, *WT* wild type.

The audit was conducted on 23rd November 2023 and includes samples from seven NHS trust pathology laboratories serving the North East of England and Cumbria region. For the 2022–2023 period, the number of patients with an MSI-H and *BRAF* wild type CRC who are still under investigation for a germline diagnosis at the time of audit is unknown and so germline genetic testing data are incomplete.

* MIP amplicon sequencing according to Gallon et al. [23]; MSI fragment length analysis (FLA) by MSI Analysis System v1.2 (Promega); *BRAF* analysis by MassARRAY System (Agena).

** Including only CRC diagnoses with histological confirmation of tissue invasion and, therefore, tissue available for molecular testing.

*** Two CRC patients from the 2022–2023 audit period were excluded from the germline genetic testing statistics. The first patient had multiple CRCs, their first being tested in the 2021–2022 period and the second in the 2022–2023 period. Both were MSI-H and *BRAF* wild type and so germline genetic testing was triggered by the first CRC in 2021–2022. The second patient was 31 years old at diagnosis of a rectal cancer and was immediately referred for germline genetic testing and CRC testing was only requested subsequent to germline genetic testing.

To assess potential impact on LS screening, comparisons were made to the same time period of two earlier years when different tumour tests were used (Table 1). In 2019–2020, a multi-test pathway of MSI FLA (Promega) followed by *BRAF* analysis by MassARRAY (Agena) was used. In 2021–2022, MIP amplicon sequencing [23] was primarily used along with the multi-test pathway for low quality/quantity samples ( ~ 14% of cases). The total regional number of CRC diagnoses uses histologically-confirmed CRC patients, therefore excluding CRCs diagnosed through scans or other modalities where tissue was not available for testing.

In 2022–2023, the Newcastle MSI-Plus Assay tested 97.2% (2466/2536) of histologically-confirmed CRC patients, slightly more than the 95.1% tested in 2021–2022 (2236/2351; *p* = 1.2 × 10^–4^) and much more than the 28.6% tested in 2019–2020 (601/2104; *p* < 10^–15^) (Table 1). In 2022–2023, DNA extraction and molecular testing (including both MSI and *BRAF* analyses) were completed in a median 6 working days, from tissue sample receipt to reporting of a successful (QC pass) test result. This was quicker than the median 8 and 12 working day turnaround times achieved in 2021–2022 and 2019–2020, respectively (both *p* < 1.0 × 10^–15^). Furthermore, a lower proportion of samples failed testing in 2022–2023 (1/2565; 0.04%) compared to 2021–2022 (7/2350; 0.30%; *p* = 0.03) and 2019–2020 (10/622; 1.61%; *p* = 6.9 × 10^–7^), suggesting the new assay is more robust.

Among tested patients, the frequency of MSI-H, *BRAF* wild type CRC patients at-risk of LS was equivalent in 2022–2023 (139/2466; 5.6%) and 2021–2022 (122/2236; 5.5%; *p* = 0.52), but was higher in 2019–2020 (61/601; 10.2%; *p* = 5.1 × 10^–4^) (Table 1), likely due to more selective testing. However, the frequency of at-risk patients identified relative to total histologically-confirmed CRC patients was significantly higher in 2022–2023 (139/2536; 5.5%; *p* = 1.6 × 10^–5^) and in 2021–2022 (122/2351; 5.2%; *p* = 1.1 × 10^–4^), when either iteration of the amplicon sequencing assay was used, compared to 2019–2020 (61/2104; 2.9%), when the multi-test pathway was used.

Statistics for germline genetic testing of at-risk patients in 2022–2023 are incomplete as patients were still in the germline testing pathway at the time of audit. For the earlier periods of 2021–2022 and 2019–2020 for which data is assumed to be complete, the proportion of at-risk patients receiving a germline genetic test (respectively, 54/122; 44.3%; and 25/61; 41.0%; p = 0.75) and the frequency of LS diagnoses (respectively, 17/54; 31.5%; and 7/25; 28.0%; *p* = 0.80) were both similar (Table 1), indicating that the improved detection of at-risk patients using MIP amplicon sequencing was not associated with improved downstream germline genetic testing. A trend for more LS diagnoses among histologically-confirmed CRC patients was observed in 2021–2022 (17/2351; 0.7%) compared to 2019–2020 (7/2104; 0.3%; *p* = 0.10) (Table 1).

The regional audit had limitations (see methods), and the observed increase in CRC testing will reflect both implementation of the assay and the efforts of the regional LS Champion appointed through “The English National Lynch Syndrome transformation project” [35]. Therefore, the regional audit was complemented by an audit of high quality testing data from the Northumbria Healthcare Trust, a trust within the region that had adopted NICE Diagnostics Guidance 27 at the time of publication in 2017 and is, therefore, influenced less by the recent, national drive to improve LS screening. In the trust, 287/299 histologically-confirmed CRCs (96.0%) were tested by the MSI-Plus assay in 2022–2023, equivalent to testing in 2021–2022 using MIP amplicon sequencing (331/336; 98.5%; *p* = 0.76), and a significant increase compared to 2019–2020 using the multi-test pathway (239/279; 85.7%; *p* = 1.6 × 10^–5^). Though not statistically significant, testing identified more at-risk patients in 2022–2023 compared to 2019–2020 (respectively, 15/299; 5.0%; and 8/279; 2.9%; *p* = 0.21; Supplementary Table S4). Therefore, the high quality, trust-level data confirmed that the Newcastle MSI-Plus Assay was associated with increased testing within an already well-performing trust.

## Discussion

The Newcastle MSI-Plus Assay uses one-step multiplex PCR amplification of MSI markers and *BRAF*, *KRAS*, and *NRAS* mutation hotspots to generate a sequencing library within a few hours. Amplification from <1 ng of sample DNA make it suitable for low quantity samples, such as colonoscopic biopsies for pre-surgical LS screening and to inform the (increasing) use of neoadjuvant therapy [36]. MSI and mutation hotspot status with supporting QC metrics are provided in a single report along with clinical recommendations for LS screening and oncological management. The assay’s simplicity and automated analysis allows one full-time-equivalent genetic technologist to provide testing and reporting for a regional population of 3 million people with ~2500 CRC diagnoses per annum. Service achieved a median 6 working days from tissue sample receipt to result reporting and near complete testing of histologically-confirmed CRCs. In-house estimation priced the assay at £50-70 per sample at the time of writing, which includes all costs from reagents and consumables to staff time and overheads. The relatively low cost and accurate assessment of multiple biomarkers suggest the MSI-Plus assay will be highly cost-effective. A comprehensive health economic analysis is ongoing.

Implementation of the assay was associated with improved testing rates and turnaround times compared to previous years using either MIP amplicon sequencing or a multi-test pathway. This improvement will be related to several key advantages, such as its low cost, scalability, clarity of results, and delivery by technologists rather than senior scientists or pathologists, that address the previously identified barriers to LS screening [11–13]. However, it has also been shown that LS screening quality improvement programmes that focus on educating healthcare providers and connecting clinical services, rather than employing new technologies, significantly increase tumour testing [37]. Therefore, the improved testing observed here at the regional-level will also reflect the impact of the English National Lynch Syndrome transformation project, which has appointed over 200 local LS Champions across England and established a national forum and data service to monitor LS screening, among other advances [35]. Of note, LS Champions educate pathologists, who are critical for referral, on the importance of reflex MMR deficiency testing and the available testing pathways. In this study, the Newcastle MSI-Plus Assay was associated with improved LS screening in the Northumbria Healthcare Trust, in which NICE Diagnostics Guidance 27 had been prioritised since 2017. Therefore, the assay is highly relevant to national initiatives to improve LS screening, and it is currently being validated in other NHS England Genomic Laboratory Hubs to extend its use to other regions.

There are barriers to LS screening beyond tumour testing [38] and the impact of the assay on LS diagnoses will be limited by attrition downstream in the pathway. For example, in the NDRS audit of LS screening across England in 2019, only 28% of patients at-risk of LS based on tumour testing had a germline genetic test [14]. This is slightly lower than the 41-44% observed here for the 2019–2020 and 2021–2022 audit periods where we had complete germline testing data. The limitations caused by these downstream barriers was also apparent in this study given the lack of a significant increase in the rate of LS diagnoses in 2021–2022 from 2019–2020 despite a dramatic uptake in tumour testing. These downstream barriers are also being targeted by the English National Lynch Syndrome transformation project [35], including updates to molecular reporting to ease clinical interpretation, education of multi-disciplinary teams on results interpretation and germline testing referral, and the mainstreaming of genetic testing. As shown for increased MMR deficiency testing rates, such programmes have increased referrals for germline analysis [37].

The Newcastle MSI-Plus Assay may also facilitate testing in additional types of tumour and sample. In 2020, NICE published Diagnostics Guidance 42 recommending LS screening in all EC patients using IHC and *MLH1* promoter methylation testing [39]. MSI analysis is not recommended as it may be less sensitive than IHC in ECs especially for the detection of MSH6 deficiency [40, 41], and *BRAF* c.1799T>A variants are rare and do not discriminate between LS and sporadic MMR deficient tumours [42]. However, it may still be of interest to assess the MSI-Plus assay for EC testing, requiring validation against IHC and a tertiary method such as MMR gene sequencing, as it could resolve uncertain IHC results and detect the ~5% of MMR deficient cases caused by inactivating variants that retain protein expression [43–47]. In other LS spectrum tumour types, MMR deficiency testing without secondary tumour tests may be a viable LS screening strategy, such as urothelial and small bowel cancer following estimated positive predictive values > 25% with negative predictive values > 99% [38]. This could be facilitated by the Newcastle MSI-Plus Assay as its MSI markers appear to be tissue agnostic, with accurate detection of constitutional MSI in non-neoplastic tissues of CMMRD patients [30, 48] and MMR deficiency in LS urothelial cancers [49]. MMR deficiency testing of other tumour types also informs ICI therapy [9, 50]. In a proof-of-principle study, we also detected MSI-H, tumour-derived cell free DNA in the urines of an LS EC patient and an LS urothelial cancer patient [51]. Exploration of the assay for pan-cancer testing, for CMMRD screening, and for LS genitourinary tract cancer surveillance is, therefore, warranted.

There are limitations to this study. The new assay has only been validated on a MiSeq to date, though validation on larger scale Illumina sequencers is underway. Where resource permits, tumour testing by gene panel (or more comprehensive) sequencing may be preferable, to include other genetic markers and identify biallelic somatic MMR variants in patients who would otherwise be suspected of LS [41, 52, 53]. However, the quantity and quality of DNA extracted from biopsies continues to be a challenge for such methods. Finally, the clinical utility of the assay was demonstrated in a regional setting following UK guidelines. Therefore, aspects of this study will not be applicable to other regions and countries, although the assay’s simple methodology and other potential applications will make it of interest in a broad range of healthcare settings.

In conclusion, the Newcastle MSI-Plus Assay provides a single, low cost, scalable, and fully automatable tumour test for LS screening and oncological management of CRC patients that was associated with improved LS screening across a regional population of 3 million people.

### Supplementary information

Supplementary information

Supplementary table

## Data Availability

Amplicon sequence FASTQ files are available from the European Nucleotide Archive (https://www.ebi.ac.uk/ena/browser/home) using study ID PRJEB67820.

## Code availability

R (version 4.2.2) code for simulation analyses is available on request from the corresponding author.
