# Supplementary material for: A novel colorectal cancer test combining microsatellite instability and BRAF/RAS analysis: Clinical validation and impact on Lynch syndrome screening
Source: BJC Rep. 2024 Jul 1;2:48. doi: 10.1038/s44276-024-00072-8 (PMC11216981; doi:10.1038/s44276-024-00072-8)
Supplement: Supplementary file 1 — Supplementary information [file 44276_2024_72_MOESM1_ESM.pdf]

# Supplementary Figures

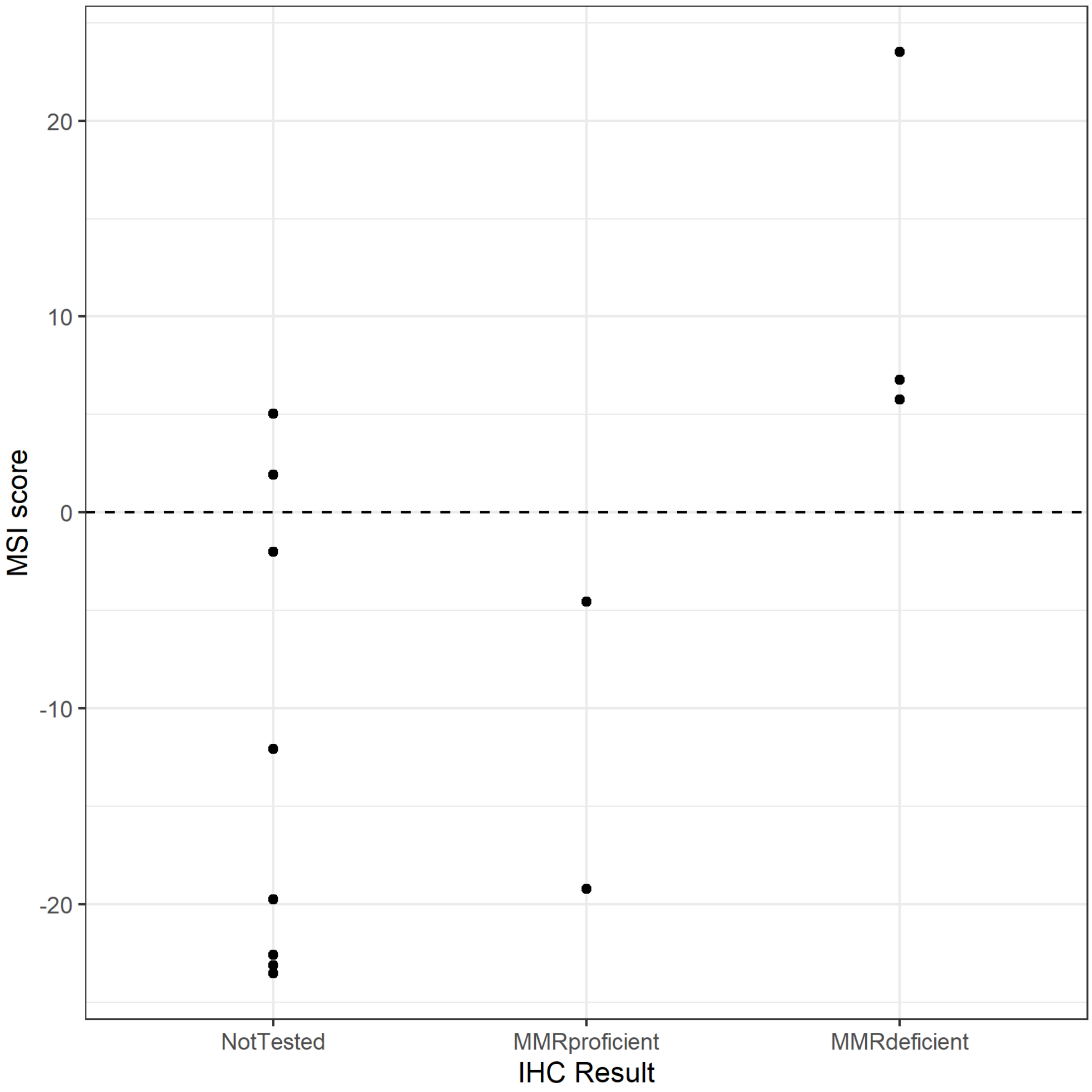


**Supplementary Figure S1.** Comparison of MSI score (Newcastle MSI-Plus Assay) and mismatch repair (MMR) protein expression by immunohistochemistry (IHC) for 13 CRCs that were classified as MSI-H based on instability in 2/5 markers using the MSI Analysis System v1.2 (Promega). For CRCs analysed by all three methods, the results show that 40% (2/5) of the CRCs classified as MSI-H (Promega) have MSI scores <0 indicating they are MSS (Newcastle MSI-Plus Assay) and are MMR proficient (IHC), suggesting the original MSI-H classification based on instability in 2/5 MSI markers was incorrect. Note: MSI Analysis System v1.2 testing did not have matched normal DNA samples to exclude germline variants, which may account for these misclassifications. Therefore, for the validation of the Newcastle MSI-Plus Assay, samples with an MSI-H reference classification based on instability in 2/5 MSI markers by the MSI Analysis System v1.2 were excluded, unless an alternative reference result using IHC was available.

Supplementary Figures

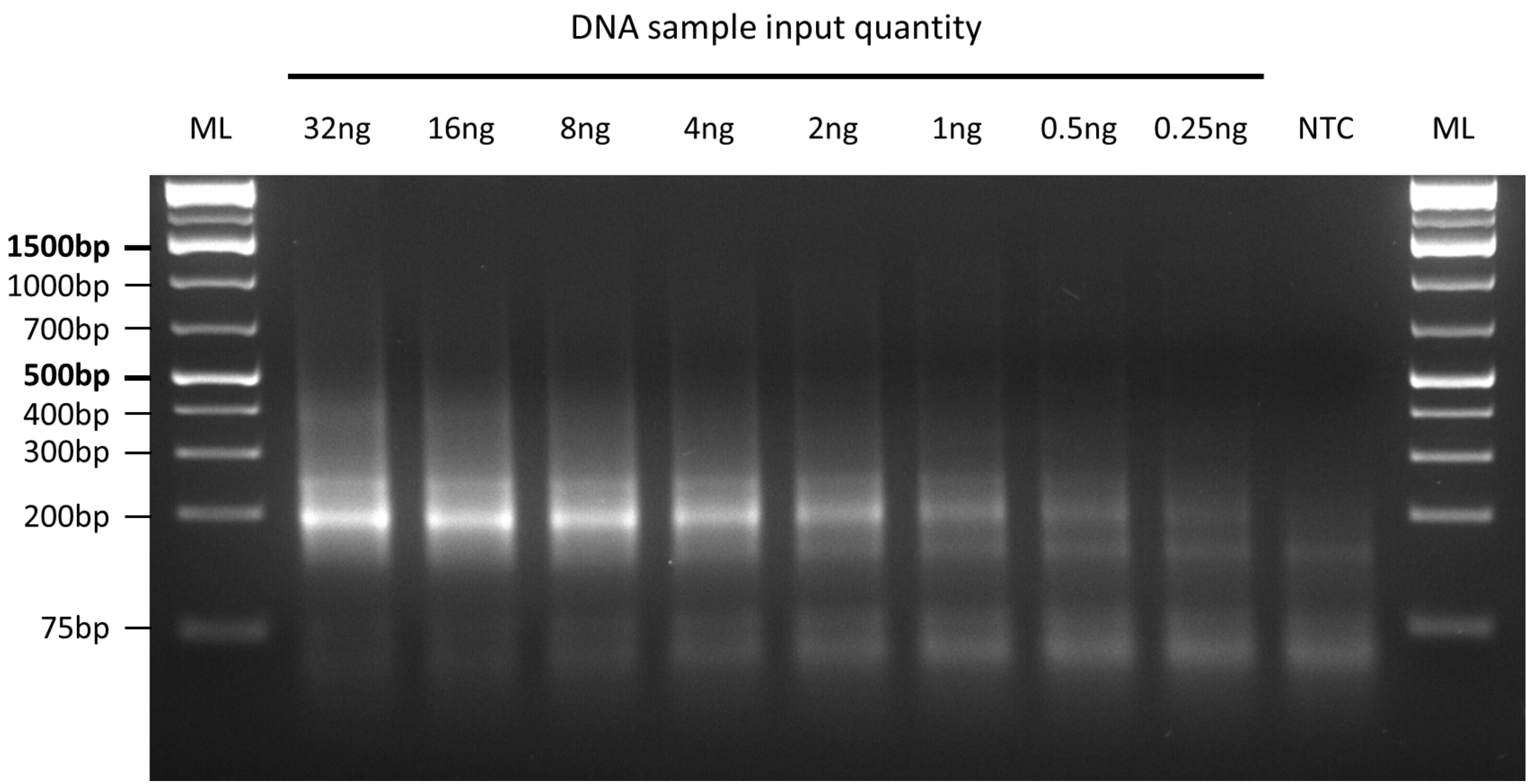


**Supplementary Figure S2.** Amplicons generated by the Newcastle MSI-Plus Assay from a serially diluted peripheral blood leukocyte genomic DNA sample, from 32ng to 0.5ng, visualised using 3% agarose gel electrophoresis and ethidium bromide fluorescence under UV light. Target specific amplicons are 189-258bp and are evident with as little as 0.25ng of template DNA. A relatively large primer dimer is evident at ~150bp due to the relatively long primers used (74-83bp). The marker ladder (ML) is GeneRuler 1 kb Plus Ladder (ThermoFisher Scientific). NTC = negative template control.

# Supplementary Figures

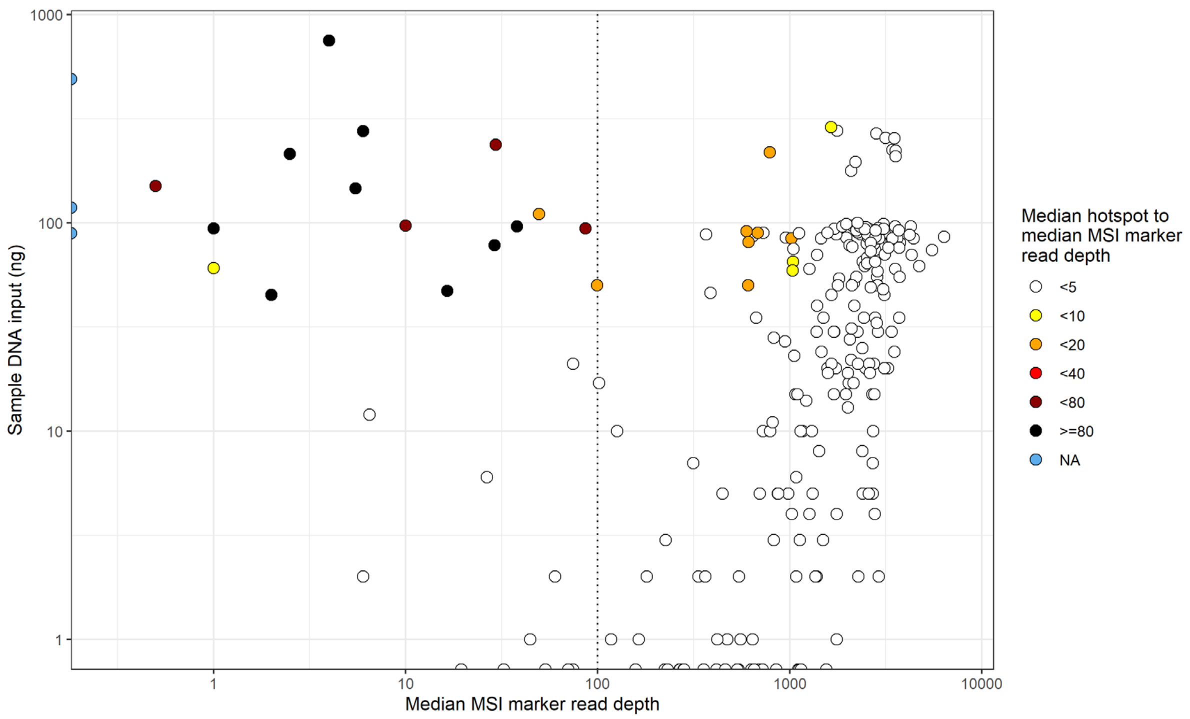


**Supplementary Figure S3.** Reaction input quantity of DNA compared to median MSI marker read depth of training and validation cohort samples analysed by the Newcastle MSI-Plus Assay. Data point colour represents the ratio of median mutation hotspot to median MSI marker read depth, with darker colours representing higher ratios.

## Supplementary Figures

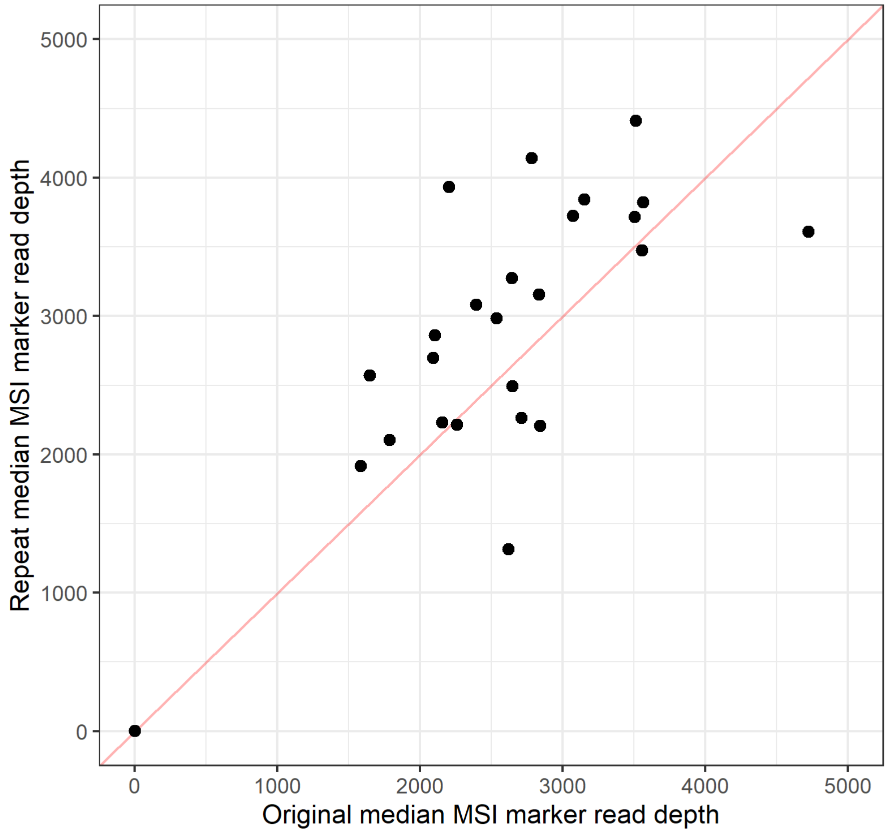


**Supplementary Figure S4.** Comparison of median MSI marker read depths between repeat and original assays of 25 CRCs from the validation cohort using the Newcastle MSI-Plus Assay.

## Supplementary Figures

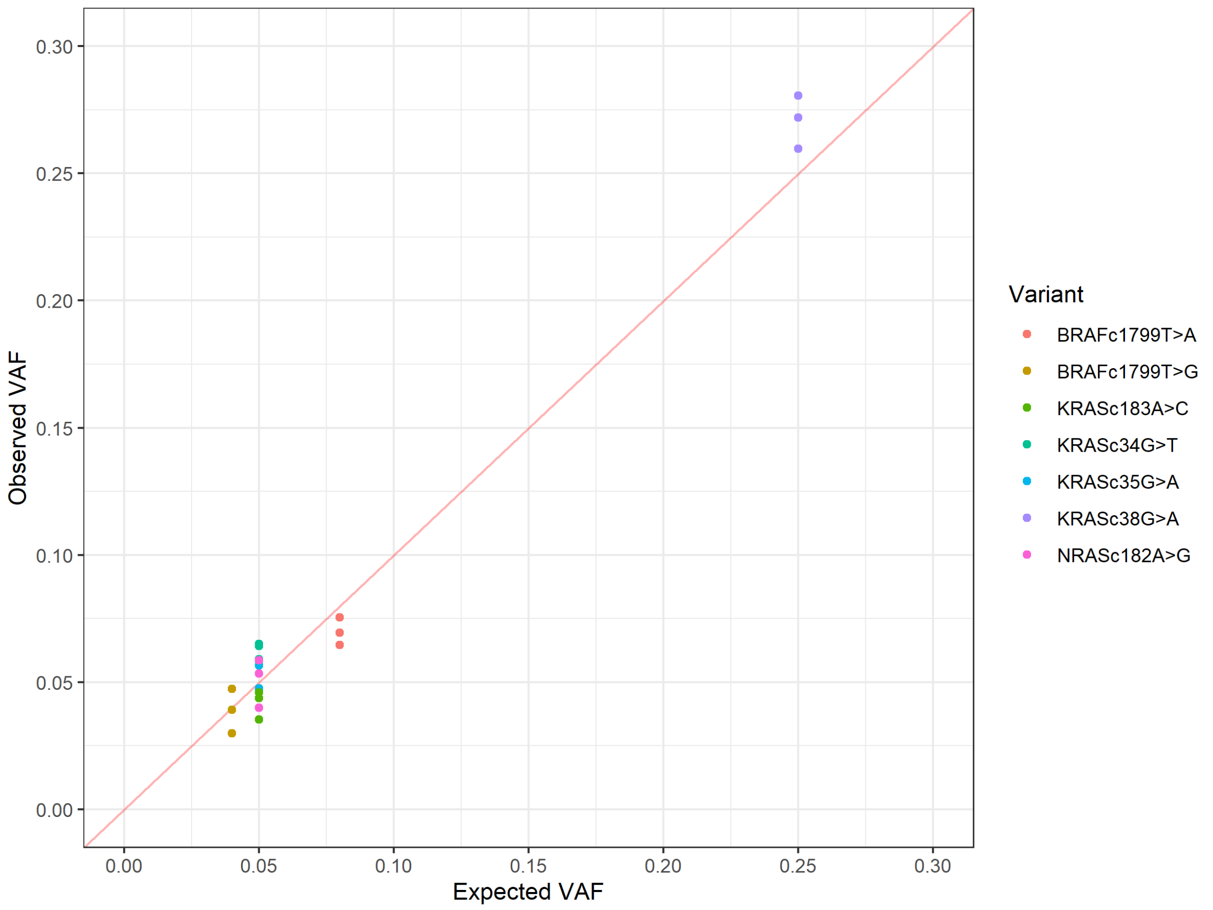


**Supplementary Figure S5.** The observed variant allele frequencies (VAF) of seven *BRAF*, *KRAS*, and *NRAS* variants detected by the Newcastle MSI-Plus Assay compared to the expected VAF for the Tru-Q 4 (5% Tier) Reference Standard (Horizon) assayed in triplicate.

# Supplementary Figures

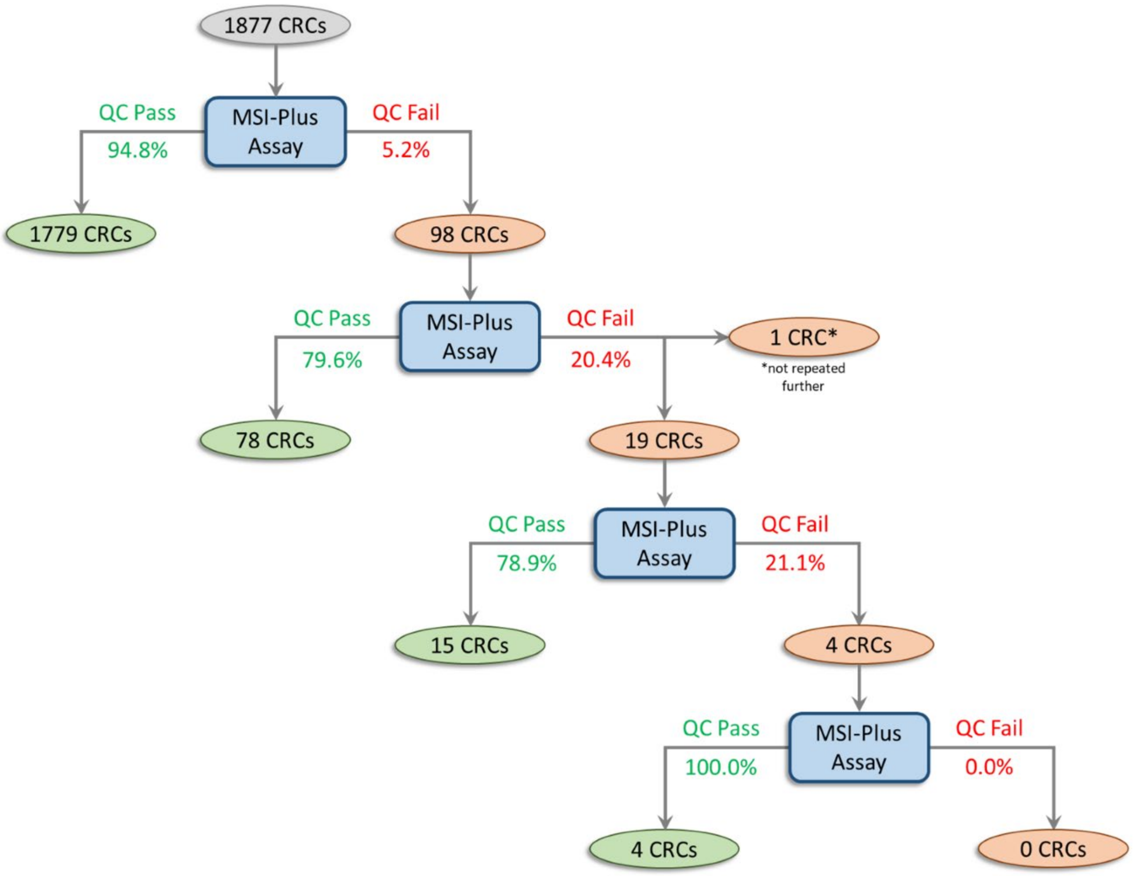


**Supplementary Figure S6.** The frequency of test QC failures and repeat tests between January 18th 2023 and October 17th 2023. Note, only the last 9 months of the first year audit are shown for two reasons. One, repeat tests were systematically recorded only from January 2023 – clear data on the number of repeat tests were readily available for the last 9 months of the audit. Two, after clinical validation, the assay was immediately deployed into clinical service but optimisation of the multiplex PCR primer concentrations was needed to ensure sufficient reads for the *BRAF* amplicon – data from the last 9 months of the audit excludes the primer optimisation period to give a fairer representation of test QC failure rate.
